# WiGAN: A WiFi Based Gesture Recognition System with GANs

**DOI:** 10.3390/s20174757

**Published:** 2020-08-23

**Authors:** Dehao Jiang, Mingqi Li, Chunling Xu

**Affiliations:** 1 Shanghai Advanced Research Institute, Chinese Academy of Sciences, Shanghai 201210, China; jiangdh@shanghaitech.edu.cn (D.J.); xucl@sari.ac.cn (C.X.); 2 School of Information Science and Technology, ShanghaiTech University, Shanghai 201210, China; 3 University of Chinese Academy of Sciences, Beijing 100039, China

**Keywords:** wireless, gesture recognition, channel status information, generate adversarial network, support vector machine

## Abstract

In recent years, a series of research experiments have been conducted on WiFi-based gesture recognition. However, current recognition systems are still facing the challenge of small samples and environmental dependence. To deal with the problem of performance degradation caused by these factors, we propose a WiFi-based gesture recognition system, WiGAN, which uses Generative Adversarial Network (GAN) to extract and generate gesture features. With GAN, WiGAN expands the data capacity to reduce time cost and increase sample diversity. The proposed system extracts and fuses multiple convolutional layer feature maps as gesture features before gesture recognition. After fusing features, Support Vector Machine (SVM) is exploited for human activity classification because of its accuracy and convenience. The key insight of WiGAN is to generate samples and merge multi-grained feature maps in our designed GAN, which not only enhances the data but also allows the neural network to select different grained features for gesture recognition. According to the result of experiments conducted on two existing datasets, the average recognition accuracy of WiGAN reaches 98% and 95.6%, respectively, outperforming the existing system. Moreover, the recognition accuracy under different experimental environments and different users shows the robustness of WiGAN.

## 1. Introduction

With the rapid development of virtual reality and smart home, human–computer interaction applications are becoming more and more popular in our life. Since human gesture recognition can improve the quality of human–computer interaction and intelligent services, it has become one of the most important research hotspots in intelligent applications.

Traditional gesture recognition methods are mainly based on cameras [1], wearable sensors [2], RFID [3], radar [4], and other special equipment [5]. Compared with these methods, WiFi-based gesture recognition methods are of more importance. For example, cameras have advanced technology and high accuracy in recognizing human gestures; however, these devices are usually expensive and there is a risk of privacy leakage. In contrast, WiFi-based gesture recognition has the superiority of low cost, no special equipment, scanning through walls, and privacy protection, which is better than traditional methods in certain aspects. Therefore, the demand for human–computer interaction has led to extensive research on gesture recognition using commercial WiFi devices.

In general, signal indicators used in WiFi-based gesture recognition are mainly received signal strength (RSS) and channel state information (CSI). Due to the convenience of collecting data, RSS [6,7] is often used in scenarios with simple actions and low equipment requirements. However, when the distance increases and the multipath effect becomes obvious, the performance of RSS will be significantly reduced in some complex environments. Therefore, with the deepening research, researchers prefer to use CSI instead of RSS to recognize human gestures. Compared with RSS, CSI greatly improves the accuracy of gesture recognition because it can provide more information. At present, some CSI-based works such as E-eyes [8], WiFall [9], WiMU [10], and TW-See [11] have been proposed, which shows that WiFi-based gesture recognition has become an important part of the state-of-the-art. E-eyes collects CSI information on commercial WiFi devices to recognize nine typical daily in-place activities and eight walking activities under two environments. On the basis of motion detection by anomaly detection algorithm, WiFall, which is designed by using the temporal stability and frequency diversity of CSI applies a one-class Support Vector Machine classifier and Random Forest algorithm to achieve fall detection. WiMU further segments DFS power profile for multi-person activity recognition. TW-See is a device-free wireless recognition system that can recognize human activities passing through walls, which uses an Or-PCA approach to obtain the correlation between human activities and corresponding changes of CSI. TW-See extracts features based on correlation and uses neural networks to realize activity recognition.

However, most of the current research is carried out in the experimental environment. In practical applications, we often encounter some problems which are mainly caused by imbalanced sample classes and inconvenient movement collection. In this paper, the situation of limited training samples is referred to a small sample problem. For commodity devices, they need to collect data to learn and recognize gestures as soon as the user uses them, but each class of samples collected is usually imbalanced. For example, the WiKey [12] proposed by Ali et al. is a classic human–computer interaction system, which can perform key recognition based on the CSI changes caused by the subtle movements of human fingers during typing. However, it is non-uniform for the usage rate of each key, which results in a class imbalance problem in offline training. In addition, in the scenario of fall recognition [13], it is difficult to collect falling data because the fall action occurs with a small probability. However, we usually need a lot of data to ensure system performance in these scenarios. In addition, if the fall data are forcibly collected, there will be a high time cost. Therefore, these small sample problems have affected the popularity and application of gesture recognition algorithms. Moreover, the experimental results of most existed systems depend on specific experimental environments and fixed users, which will significantly reduce the performance of cross-domain recognition [14,15]. In this paper, we refer to this phenomenon of not adapting to new scenes as environmental dependence. This is a relatively difficult problem to address in gesture recognition, and each researcher has a different focus. In recent years, many researchers have been striving to make their systems adaptable in some aspects. For example, WiFinger [16] devises an environmental noise removal mechanism to mitigate the effect of environmental changes. WiAG [17] presents a novel configuration estimation scheme that automatically identifies the position and orientation of the user, which enables position and orientation agnostic gesture recognition.

**Figure 1:**
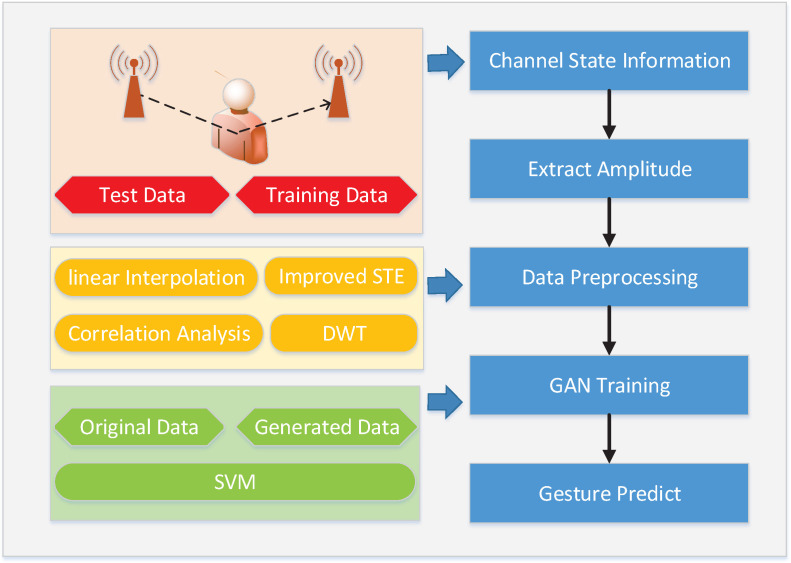
The flow of our gesture recognition.

To tackle the above two problems, this paper proposes a CSI-based gesture recognition algorithm, namely WiGAN. The system does not require any additional equipment or wearable sensors to complete gestures recognition, thus greatly reducing the system cost. The gesture recognition process of our system is shown in Figure 1. First of all, since raw CSI data have a series of noise, some data processing approaches are used to remove the noise and extract the amplitude characteristics. Second, based on GAN, WiGAN not only performs data generation in the generator, but also fuses features in the discriminator. Finally, SVM [18,19] is utilized for human activity classification, which is more suitable for small-scale training samples. Through a lot of experiments, we can infer that the data enhancement by GAN increases the diversity and number of samples, meaning that the problem of small sample caused by class imbalance and difficulty in motion collection is addressed. With the increase of the sample diversity and the application of feature fusion methods [20], compared to other systems, WiGAN can better deal with the environmental dependency issue. Experimental results show that our system can effectively improve the recognition performance. The main contributions of this paper are summarized as follows:

- We propose a WiFi-based gesture recognition system, WiGAN, which addresses the problem of performance degradation caused by small samples and the environmental dependence. The idea of feature fusion and generation is presented for gesture recognition with limited samples.
- A GAN model that combines feature maps of different layers is presented, where more diverse features are extracted to recognize gestures. By taking superiority of the data enhancement and feature fusion in GAN, WiGAN not only saves the time of collecting difficult samples, but is also beneficial to dealing with small sample problems.
- Extensive experiments are conducted to show that WiGAN has a better recognition performance and it possesses good properties of robustness.

The rest of the paper is organized as follows. Section 2 provides an overview of related work. Section 3 describes the design of each module in the system. Section 4 introduces the structure and function of GAN in detail. Section 5 presents the experiments and evaluations. Finally, we conclude our work in Section 6.

## 2. Related Work

As we know, existing gesture recognition methods can be divided into two categories: device-based systems and device-free systems. Although most of the device-based systems can achieve an impressive estimation accuracy, they often employ additional equipment such as sensors, cameras, or smartphones. Instead, device-free systems work without any special equipment. Such systems are mainly based on RSS and CSI, which are more convenient than device-based systems in many scenarios. In this paper, we explored CSI-based gesture recognition because CSI contains more fine-grained information.

In Section 2.1, we will briefly describe the superiority and weaknesses of CSI-based gesture recognition methods. Since our work studies the application of GAN in gesture recognition, the differences of several CSI-based gesture recognition methods using GAN will be separately discussed in Section 2.2.

### 2.1. CSI-Based Gesture Recognition

There are three main CSI-based gesture recognition methods: model-based gesture recognition, fingerprint library matching-based gesture recognition, and learning-based gesture recognition.

Model-based methods mainly analyze the correlation between CSI dynamics and each gesture, and then establish a model to show it. The model is used to calculate parameters to finally realize gesture recognition. For instance, Wang et al. [21] built a human activity recognition system, CARM, which uses CSI-Speed and CSI-Activity models to estimate the correlation between CSI dynamics and human activities. It recognizes a given activity by matching it to the best-fit profile based on this correlation. The QGesture [22] system establishes a one-dimensional scene model and a two-dimensional scene model respectively, which can measure the movement distance and direction of human gestures in two scenarios. Model-based methods are generally simple to train models and do not require large datasets. However, they have strong environmental dependence, so it is difficult to effectively identify in new scenes.

Fingerprint library matching-based methods usually establish a complete fingerprint library about CSI features to recognize a given gesture by matching gesture features with the fingerprint library. For instance, Li et al. [23] proposed WiFinger to identify finger-grained gestures by extracting gesture features and using K-nearest neighbor and dynamic time warping (KNN & DTW) to match the fingerprint library. Al-qaness et al. [24] presented WiGeR to identify human gestures by calculating DTW distances between given samples and fingerprint library samples. Fingerprint library matching-based methods can achieve a reliable recognition accuracy, but they require sufficient samples to build a fingerprint library. In a small sample scenario, they usually cannot show better performance.

Learning-based methods usually combine signal processing algorithms and machine learning for gesture recognition. For example, Ma et al. [25] presented a sign language recognition system, SignFi, which uses a nine-layer convolutional neural network (CNN) to realize the recognition of 276 sign gestures with an accuracy of 94%. CrossSense [26] applies transfer learning to effectively reuse the learned knowledge across different sites and tasks. At present, deep learning-based gesture recognition has become a research hot spot. When the data features are inconspicuous, deep learning can learn better features compared with other methods. The biggest obstacle for WiFi-based gesture recognition in many applications is to find a suitable feature set. However, for deep learning, the biggest excellence is that a better feature set can be learned as long as there are enough layers. Neural networks can find and characterize the complex structural features within the problem, so they greatly improve performance.

The above systems always have weaknesses that affect further popularity. Therefore, we propose a GAN-based WiFi gesture recognition system to overcome the above problems. Experiments show that WiGAN has better recognition accuracy for different environments and users under small sample conditions.

### 2.2. GAN Data Enhancement-Based Gesture Recognition

To the best of our knowledge, there are two GAN systems in CSI-based gesture recognition being AF-DCGAN [27] and CsiGAN [28]. Li et al. [27] use the AF-DCGAN model to generate more amplitude feature maps of the sampling point position. It saves a great deal of time collecting each single sample point as well as human cost on the indoor positioning problem. CsiGAN is a semi-supervised learning model to address the performance degradation of leave-one-subject-out validation for CSI-based activity recognition. In contrast, WiGAN is primarily a gesture recognition system specialized on the condition of small samples. The system pays more attention to address the problem of small samples and environmental dependencies. Therefore, based on our GAN model, the CSI data processing module and SVM are designed to help it address these problems. To evaluate the performance of the system, we set up two datasets for comparison, and better demonstrate the superiority and robustness of the system compared to others. Moreover, unlike AF-DCGAN and CsiGAN that only use GAN to generate samples, the GAN module we designed is not only used to generate data but also extracts key layer information for fusion. This approach makes full use of coarse-grained and fine-grained features, which is equivalent to exploiting different levels of description of the same action to recognize gestures.

In addition, WiADG [15] is also a system with adversarial networks as the core. It trains a target encoder to map the target data to the domain invariant latent feature space to minimize the domain discrepancy distance between the source domain and the target domain. After that, this system uses the source domain classifier to classify target domain data to achieve cross-domain recognition. Structurally, WiGAN adds a generation module and feature fusion module compared to WiADG, which leads to a completely different theory between the two systems. Unlike using an encoder for domain mapping in WiADG, WiGAN exploits the GAN that has been trained in the source domain to enhance the data in the target domain and fuse sufficient features. Our system can also deal with the performance degradation caused by environmental dependence. WiADG conducted experiments in a conference room and an office zone to illustrate the domain adaptability of the system to the environment. In addition, we not only discuss the environmental impact but also explain the performance of the system under different users, both of which make our evaluations more extensive than previous works.

## 3. System Design

In this section, we will explain the main structure and function of WiGAN.

### 3.1. Overview of WiGAN

**Figure 2:**
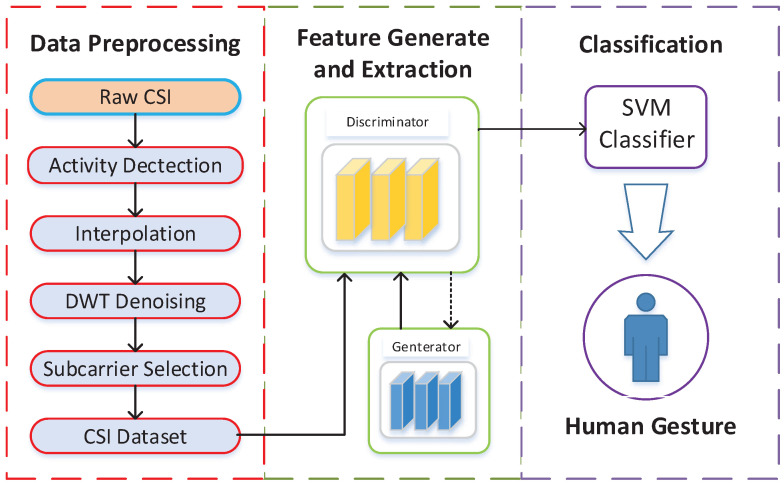
WiGAN system architecture.

WiGAN is a device-free system that can recognize gestures using commercial WiFi devices. As shown in Figure 2, this system consists of three sections: (1) Data processing; (2) Feature generation and extraction; and (3) Gesture classification. Next, we introduce the components and functions of each module.

The CSI processing module converts the raw CSI data into sanitized CSI amplitude through some signal processing approaches. Therefore, this module is mainly divided into four sections: (1) Activity detection. Extract the amplitude of CSI and cut out the gesture data in CSI. (2) Interpolation. Use linear interpolation to unify the CSI shape and compensate for CSI packet loss. (3) DWT denoising. Remove high-frequency noise in CSI. (4) Subcarrier selection. Choose subcarriers suitable for feature extraction. In general, the CSI signal processing module extracts appropriate CSI gesture data to prepare for the subsequent part.

The feature generation and extraction module is composed of GAN and CNN algorithms. In GAN, the generator is responsible for generating the preprocessed data, while the discriminator is used to extract features and classify the CSI data by softmax. In summary, GAN has played a role in enhancing data and extracting features in the system. The CNN algorithm is part of the discriminator, which has the function of reducing the feature map dimension and fusing the feature information of selected layers in the discriminator. This allows the CSI features of different grains to be combined into a complete feature set as softmax layer input for classification.

The classification module completes the recognition of gestures. Under the condition of small samples, WiGAN uses the generated data to enhance the dataset for classifier training, and it recognizes a given activity by the trained SVM classifier.

### 3.2. Channel State Information

In an indoor environment, WiFi signals propagate from the transmitter to the receiver through multiple paths, which carry environmental information during the propagation process. Therefore, we take the information that can describe the channel condition as the basic information of environmental perception, such as RSS and CSI. However, RSS measures the effect of WiFi signal multi-path propagation superposition, and cannot distinguish multiple propagation paths of signals. These weaknesses not only affect environmental perception, but also limit the further development of RSS.

Researchers began to use the Channel Impulse Response (CIR) [29] of the wireless channel to describe the channel in the time domain. However, accurate CIR cannot be extracted from ordinary wireless devices. Therefore, we convert the CIR to the frequency domain by FFT, and characterize the multipath by the channel frequency response (CFR) [30]. Finally, CFR can be obtained in the form of CSI even on ordinary WiFi devices. The received CSI signal is a matrix of $N_{t}\times N_{r}\times N_{c}$. (number of transmitting antennas × number of receiving antennas × number of subcarriers).

### 3.3. Activity Detection

**Figure 3:**
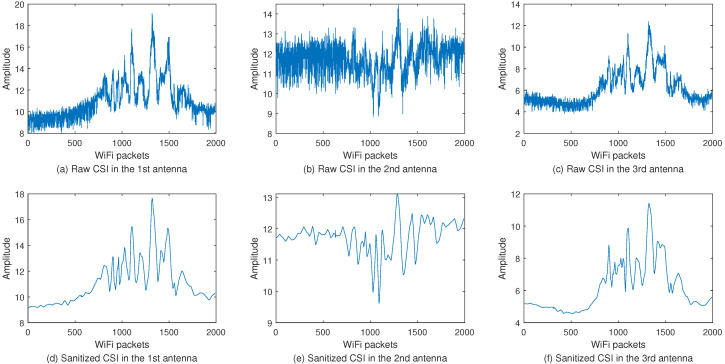
Raw CSI and sanitized CSI of the first subcarrier in each antenna. (**a**–**c**) are the CSI amplitudes obtained by collecting the same gesture signal by the three antennas of the WiFi device. (**d**–**f**) are their sanitized CSI amplitudes.

From Figure 3, we can see that there is always a small smooth segment at the beginning and end of the signal, which are static CSI data that do not contain action information. Since the presence of static CSI will interfere with gesture recognition, it is important to use appropriate approaches to segment activity data. To detect endpoints in different gestures, we propose the improved short-term energy (STE) [24] algorithm for activity recognition. The process of improved STE is divided into three sections:

(1) As shown in Figure 3a–c, the difference in CSI between different antennas is relatively large, so we consider cutting activity data on each antenna. However, each antenna contains $N_{c}$ subcarriers that have a strong correlation with others. Therefore, the algorithm first performed Principal Component Analysis (PCA) on each subcarrier of the three antennas to extract the main information of the CSI. Since the information of all subcarriers is fully utilized, the CSI information selected by using PCA is more representative.

(2) When there is no moving object around, the amplitude of CSI remains relatively constant. However, the CSI amplitude will be significantly distorted with the moving human body because human bodies are good reflectors of wireless signals. Therefore, we designed improved STE as an algorithm based on an adaptive window, by calculating the energy of the CSI value in each time window to determine whether there is activity. Generally, high energy and low energy represent the presence and absence of activity. An improved STE algorithm is designed to adjust the window length within the threshold to suit the length of activities. The short-term average energy of a speech signal at time *n* is shown as Equation (1):

(1) $$E_{n}=\sum_{m=n-(N-1)}^{n}{\left[CSI_{pca}\left(m\right)w\left(n-m\right)\right]}^{2}$$

In (1), N is the window length, $CSI_{pca}$ is the first principal component signal after PCA of CSI; w(n) is the window function, and an adaptive length rectangular window is used in this paper.

(3) Finally, we take the sum of the three window ranges and cut three antennas uniformly in the synthesis window. Under the condition of significant activity, using the obtained window to perform activity detection on all subcarriers has obtained satisfactory results.

### 3.4. Interpolation

For different gestures, the time to complete the gesture is different, which results in a different frame length for each CSI trace. To input the same shape CSI to neural networks for training, unifying the shape of the CSI is a necessary step. From this perspective, linear interpolation is a suitable method to ensure that each gesture has the same number of WiFi packets. Moreover, in the indoor environment, the link signal is significantly weakened and part of the WiFi packets will be dropped due to non-line-of-sight connections and wall penetration. Therefore, it is necessary to use linear interpolation to reasonably compensate for lack of data and adjust the CSI shape. Specifically, we first use the timestamp of each CSI to locate the CSI value, and then obtain CSI at equal intervals on the time axis according to the length of time, thereby completing the unification of the CSI shape.

### 3.5. Discrete Wavelet Transform

The CSI value describes how the amplitude of the wireless signal change when the signal travels from the sending antenna to the receiving antenna over a subcarrier. However, CSI measurements obtained from commercial WiFi devices contain noise from various sources such as interference coming from nearby devices, transmission power adaptation at the sender, and imperfect clock synchronization [31]. Figure 3a–c show the amplitude of the first subcarrier CSI of the three antennas when humans make a push action. Due to the influence of environmental noise, the fluctuation of the CSI value caused by the moving human body is irregular. Therefore, the CSI measurement value must be denoised before extracting human gesture features.

Generally speaking, the noise signal is mostly contained in higher frequency details because of the low gesture frequency. To effectively filter out noise and protect effective signals, we choose to use Discrete Wavelet Transform (DWT) for signal denoising. The first step of DWT denoising is to select the appropriate wavelet and wavelet decomposition level to perform wavelet decomposition on the signal. After signal decomposition, the decomposed wavelet coefficients are weighted by using thresholds. Finally, according to the low-frequency coefficients of the wavelet decomposition and the processed high-frequency coefficients, the signal is reconstructed by the wavelet. Compared with other noise reduction approaches, DWT denoising can protect useful signal spikes and abrupt signals, and which distinguishes detailed information from high-frequency noise. Although DWT denoising can be regarded as low-pass filtering to a large extent, but it is much smoother than the signal generated by low-pass filtering and preserves details in CSI amplitude changes. In this paper, DWT filtering uses a 5 level sym3 wavelet to decompose the signal. Through careful parameter selection, DWT filtering eliminates in-band noise, retains high-frequency components, and reduces signal distortion. The raw CSI denoising results are shown in Figure 3d–f.

### 3.6. Subcarrier Selection

When performing gesture recognition in a complex environment, the reflection of obstacles between STA and AP not only weakens the signal strength, but also brings more noise. Therefore, to achieve the purpose of ensuring recognition accuracy in some complex environments, placing multiple sets of transceiver links to collect more information is an excellent solution. However, the system processing too much data at one time will slow down the running speed. From [13,23,32], it can be known that the CSI power segments of different subcarriers have correlations. In addition, when humans move, the correlation becomes more obvious. Therefore, we follow the method in [32] to apply this principle to select subcarriers. To remove insensitive subcarriers to activity, it is necessary to calculate the correlation between subcarriers and select subcarriers with high correlation changes. In this way, WiGAN tries to select subcarriers that can represent changes in motion.

### 3.7. Generative Adversarial Network

The Generative Adversarial Network [33,34] is a deep learning model, which consists of two modules, namely the generator (G) and the discriminator (D). G captures the distribution of sample data and generates fake samples to deceive D, and D competes with G by distinguishing between real samples and false samples. Usually, G and D are alternately trained to achieve dynamic balance through games with each other. However, it is difficult for GAN to achieve balance through training. The objective function of GAN is shown below:

(2) $$\begin{array}{c} \min_{G}\max_{D}V\left(D,C\right)=E_{x∼P_{data}\left(x\right)}\left\{\log D\left(x\right)\right\}+E_{z∼P_{z}\left(z\right)}\left\{\log\left(1D\left(G\left(z\right)\right)\right)\right\} \end{array}$$

For WiGAN, combining the structure of Deep Convolution Generation Adversarial Network (DCGAN) [35] and the characteristics of Conditional Generative Adversarial Network (CGAN) [36], we propose a conditional convolution generation adversarial network, which can control the generation of small samples using control conditions. Compared with other GAN structures, our GAN adds a CNN module to fuse the feature maps of the last four layers in D, which is conducive to combine more abundant features for recognition.

**Figure 4:**
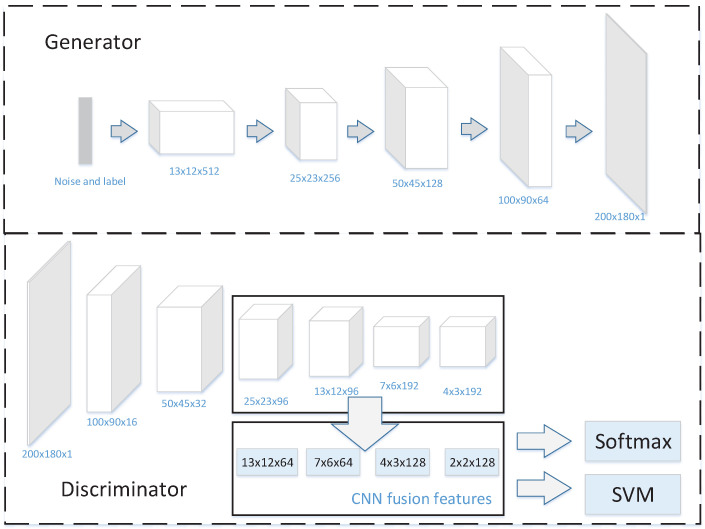
GAN structure.

To better show the performance of the system, the accuracy of WiGAN is evaluated under supervised and semi-supervised conditions. From the experiment in Section 5.3, our GAN has achieved excellent results. In supervised learning, when entering labeled samples and labels, D outputs the probability of k + 1 classes, where real samples are classified in the first k categories, and produced generated samples are classified in the (k + 1)th category. In semi-supervised learning, it is necessary to input labeled samples, labels, and unlabeled samples. For unlabeled samples, D is a binary classifier that judges the input unlabeled data as real samples or fake samples. Reflected on the output of D, the first k categories are real samples, and the (k + 1)th category is false samples. For labeled samples, they are divided into k + 1 categories like supervised learning. The structure of GAN is shown in Figure 4.

### 3.8. Classifier

The classifier is used to classify features fused by GAN. To select the most suitable classifier for small samples, we compared eight commonly used classification algorithms. According to Section 5.4.1, the best performing classification algorithm is SVM [18,19] based on radial basis function kernel (RBF). SVM is a supervised model, which maps input linear inseparable samples to a high-dimensional feature space by using a kernel function, so that original linear inseparable samples become linearly separable. This algorithm finds the largest edge hyperplane in the transformed feature space for classification.

In our research, there are two main reasons for using SVM as the final classifier. On the one hand, compared with other algorithms, SVM is suitable for the scenario with a small number of samples and imbalanced classes. Its accuracy is even slightly higher than the CNN algorithm under small sample conditions. On the other hand, the classifier needs to input generated samples and original samples at the same time, both of which are multi-layer fusion features. Compared with other classification algorithms, SVM is convenient to input multi-grained features for classification.

## 4. GAN Model Design

### 4.1. Generator and Discriminator

The generator G in WiGAN consists of a deconvolution layer, batchnorm layer, softmax, and ReLU activation function. Since the structure of DCGAN is stable, we decided to use full convolution to form G and introduce class labels in G to guide the sample generation process. In this way, G can generate data according to the label to address the small sample problem. For our discriminator D, with a similar structure to G, it adds a feature fusion module that contains four convolutional layers. Here, the feature fusion module convolves the last four layers of network features to compress information, and then inputs all features into the softmax layer to calculate the probability. Generally speaking, real samples and fake samples are put into D alternately. As shown in Figure 4, our proposed GAN model extracts the features of four convolutional layers in D and inputs them into the CNN for further convolution. In this way, the size of feature maps is reduced to facilitate SVM classification. It is worth explaining that softmax is used to classification during GAN training, while SVM does not belong to GAN. It takes the generated and original features as input to recognize given gestures. In general, D mainly plays the role of feature fusion and classification.

The discriminator D performs feature extraction through convolution. After continuous convolution, the original features are continuously concentrated, and the deeper features finally obtained make the sample classification more reliable. Just like for an image, in the feature extraction stage, the shallow network can capture some simple features of the image, such as shapes and lines. Due to the larger receptive field and more convolution operations, the deep network can capture abstract features. After extracting process features, D combines these features maps of different layers as compound features to recognize gestures.

In this way, compared with other GANs, the proposed GAN can make full use of the effective features of each layer for recognition. Since both shallow and deep networks have their focus and strength, fully integrating features of different sizes is more suitable for gesture recognition. Moreover, the convolution process is usually accompanied by the loss of important information, so feature fusion can also use as much information as possible for recognition. In short, the superiority of GAN in our system is that, no matter which depth of the feature is effective for recognition, there may be opportunities to use it. This method enables the network to learn and recognize the importance of different depth features by itself.

### 4.2. GAN Training Process

Our training process is shown in Algorithm 1. By setting cycles, GAN fixes one parameter at a time to update another ($\theta_{D}$ and $\theta_{G}$), and then iterates the process continuously until it reaches balance. From [28], we know that it is easier to collect a large amount of unlabeled data in the process of offline data training than to collect labeled data. Therefore, the semi-supervised GAN model is more suitable for practical applications. To better evaluate and draw conclusions, the two cases of semi-supervised learning and supervised learning are compared in the experiment. In semi-supervised learning, $x_{t}$ is labeled data and unlabeled data. However, we only use labeled data as $x_{t}$ in supervised learning. To easily observe the training effect, the algorithm set an evaluation breakpoint parameter M (line 2). That is, within a cycle (lines 2–5), the training accuracy and validation accuracy are evaluated every a certain period, which is beneficial to save debugging time. The training process of each epoch (line 6–13) is that G firstly generates fake samples through the input uniform noise z and label y. Then, D exploits the input fake sample and the real sample to calculate $L_{D}$ (D loss) and $L_{G}$ (G loss) (lines 8, 11) Finally, the Adam algorithm with the default parameters is used to update the generator parameter $\theta_{D}$ (line 9) and the discriminator parameter $\theta_{G}$ (line 12) until GAN reaches dynamic balance.
**Algorithm 1:** Optimization of GAN Model via the Adam Method. **Input:** Training data $x_{t}$, validation data $x_{v}$, learning rate η, label y, hyper-parameter β1, β2,ϵ, training epochs $N_{epochs}$ **Initialize:** Discriminator with parameter $\theta_{D}$, genertor with parameter $\theta_{G}$, number of batch $N_{batchs}$, uniformly distributed random vector z 1: **for** t = 0 to $N_{epochs}$
**do**
 2:  **if** i % M == 0 **then** 3:   Sample a batch of validation data $x_{v}$ and training data $x_{t}$. 4:   Calculate and save validation accuracy and training accuracy. 5:  **end if** 6:  **for** batch = 0 to $N_{batchs}$
**do**
 7:   Sample a batch of data $x_{t}$ and label y. 8:   Updata discriminator loss $L_{D}$ 9:   Use Adam algorithm to update $\theta_{D}$ by minimize $L_{D}$ 10:   Sample a batch of label y and z. 11:   Updata genertor loss $L_{G}$ 12:   Use Adam algorithm to update $\theta_{G}$ by minimize $L_{G}$ 13:  **end for** 14: **end for**


## 5. Experimental Evaluation

### 5.1. Experimental Setup

We conducted experiments on two CSI-based gesture recognition datasets and implement WiGAN in MATLAB and Tensorflow.

(1) Widar3.0 data: Zheng et al. [14] collected dozens of different gestures at 5.825 GHz using the Linux CSI Tool. Due to the excessive number of gesture samples, we extracted push, sweep, clap, and slide actions from 14 users. In addition, we use a large number of the first three actions but use only a small amount of the fourth action to create a small sample condition. The dataset samples come from three experimental environments: classroom, auditorium, and office. Each environment is divided into five gesture collection locations, and the user performs gestures in five directions at each location (details in [14]). In this dataset, each gesture uses one transmitting device and six receiving devices to collect. Since this is a complex dataset, we use the CSI processing module to finally process the CSI shape as 200×180×1.

(2) SignFi data: Ma et al. [25] used openrf modified by the 802.11n CSI tool to collect thousands of sign language gestures in CSI. With a transmission power of 15 dBm, WiFi AP and STA work under the conditions of 5 GHz and 20 MHz bandwidth. There are a total of 276 sign language gestures collected by five users in experimental environments of lab and home. Since SignFi data are relatively standard, there is rarely use data processing in the dataset. Compared with Widar3.0 data, SignFi data have too many gesture categories, which leads to the number of samples in the SignFi dataset far exceeding the Widar3.0 dataset. Therefore, the purpose of doing experiments on this dataset is to observe the recognition performance of WiGAN under conditions of large samples and multiple categories. We want to compare the performance of WiGAN on two datasets.

In the following experiment, 10-fold cross validation and user-based leave-one-subject-out validation are used to compare with other methods under different conditions. WiGAN is optimized by Adam with learning rate η = 0.0002 and the mini-batch size of data is 64. The hyper-parameters β1,β2, and ϵ use the default parameters in tensorflow. In these experiments, training and testing are performed by a Linux desktop with some GPU such as Tesla K80, Tesla K40m, GeForce GTX 1080, and TITAN Xp.

### 5.2. The Comparison between WiGAN and Existing Methods

On two datasets, WiGAN is compared with several existing gesture recognition methods (WiGeR, WiFinger, SignFi). In these methods, WiGeR [24] identified human activities by calculating the DTW distance between CSI traces and given samples; WiFinger [23] used KNN and the DTW algorithm to recognize finger-grained gesture; SignFi [25] exploited features containing both amplitude and phase, and then adopted a nine-layer CNN for sign gesture classification. In the CNN experiment, we found that, using our CSI processing method, only the CSI amplitude as input can get better results compared with SignFi. Therefore, to be consistent with our system, only the amplitude is used as input in the CNN method. Both the DTW and KNN and the DTW experiments are the same as the original system (WiGeR and WiFinger). Among these four systems, the methods used in WiGeR and WiFinger belong to fingerprint library matching-based methods, while SignFi and WiGAN use the deep learning-based methods. In the following experiment, the strengths and weaknesses of these two categories of methods are compared.

#### 5.2.1. Performance of Different Methods

**Figure 5:**
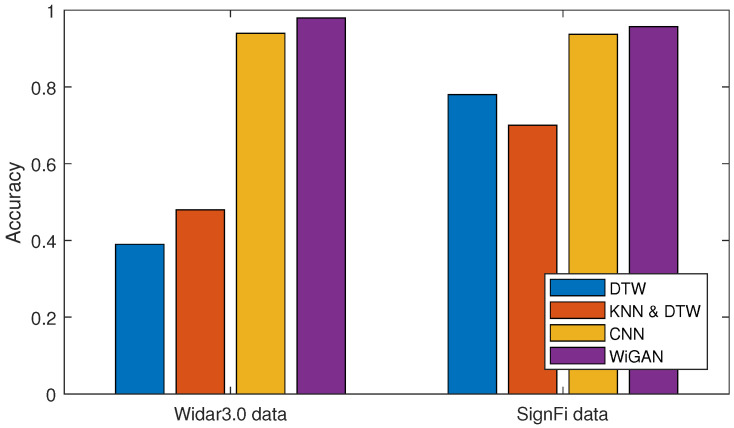
Comparison of recognition accuracy in different methods.

To demonstrate the performance of our system in gesture recognition, 10-fold cross validation is performed for four approaches on two datasets. Figure 5 shows the system performance of the four approaches, of which WiGAN achieves the best performance. In particular, WiGAN reaches 98% and 95.6% accuracy on both datasets respectively, and its performance is between 2% and 5% better than CNN. This is because data enhancement and feature fusion in WiGAN play an important role, making WiGAN generally superior to other methods under the conditions of large and small samples. Moreover, from the comparison of several methods, the recognition accuracy of WiGAN is 50% and 20% higher than those of WiGeR and WiFinger respectively on two training sets. The reason why the performance of fingerprint library matching-based methods is significantly reduced in Widar3.0 is that they cannot overcome the problems of small sample and complex environment. Therefore, the performance gap between Widar3.0 data and SignFi data is particularly obvious. From this result, compared with fingerprint library matching-based methods, WiGAN has always performed well in two scenarios.

#### 5.2.2. Performance of Different Environments

**Figure 6:**
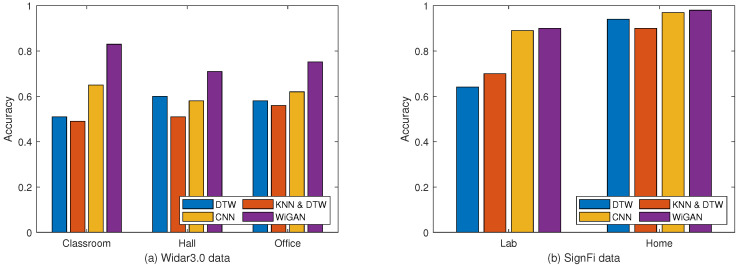
Comparison of the recognition accuracy of different environments on the two data. (**a**) Widar3.0 data. (**b**) SignFi data.

To evaluate the robustness of WiGAN in different experimental environments, 10-fold cross-validation is performed on two datasets. Figure 6 describes the recognition accuracy of the four systems in different environments. For the Widar3.0 data in Figure 6a, our system WiGAN achieves an average recognition accuracy of 83%, 71%, and 75.2% for environments ’classroom’, ’hall’, and ’office’, respectively. WiGAN performs best in all three environments, which shows that the system is suitable for different environments. For the SignFi data in Figure 6b, the recognition accuracy in the home environment is 98%, but only 90% in the lab environment. This result is only slightly better than the CNN method. As learned from [25], the major reason is that the lab has a complex multi-path environment, which heavily impacts the WiFi signal. Another reason is that the distance between the WiFi AP and STA in the lab is longer than that of the home environment, which leads to low reflection signal and more noise signals for the lab environment. It can be seen from this that the complexity of the experimental environment has a serious impact on gesture recognition.

#### 5.2.3. Performance of Different Users

**Figure 7:**
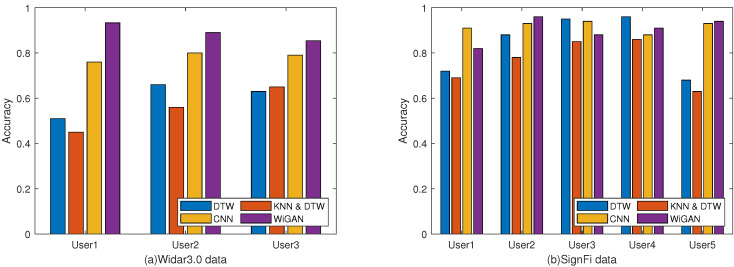
Comparison of the recognition accuracy of different users on the two data. (**a**) Widar3.0 data. (**b**) SignFi data.

**Figure 8:**
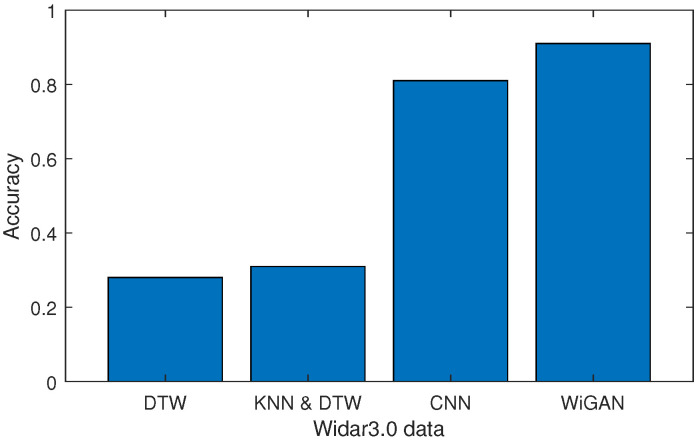
Comparison of the accuracy of cross-user recognition.

To evaluate the robustness of WiGAN for various users, 10-fold cross validation and leave-one-subject-out validation are performed on two datasets. As shown in Figure 7a, the accuracy of WiGAN is better than CNN by 5% to 10%, and is over 20% better than DTW and KNN and DTW. However, as shown in Figure 7b, the performance of WiGAN is not always the best on the SignFi dataset. The reason is that the dataset has too many categories, and WiGAN is not good at dealing with this situation. As shown in Figure 8, we conducted leave-one-subject-out validation on Widar3.0 data by cross user. Compared with the other three methods, the recognition accuracy of WiGAN is 91 %, which is better than others. That is to say, when WiGAN uses existing personnel for training and then uses new users for testing, WiGAN recognition accuracy will reach 91%. This experiment shows that WiGAN is robust and capable of addressing the performance degradation caused by environmental dependence.

### 5.3. Impact of Different GANs

In order to evaluate the performance of our GAN, the recognition accuracy of WiGAN is compared with three other GAN-based algorithms. Semi-supervised GAN (SSGAN) [37] is a popular model. When used in supervised learning, the semi-supervised part is not used in supervised learning. CGAN [36] is a kind of GAN with conditional constraints, which introduces labels to generate samples in G. DCGAN [35] and WiGAN use the same structure in this experiment, both fuse the middle layer as the feature, and finally use the softmax layer to calculate the probability. However, during gesture recognition, WiGAN will generate similar sample features to enhance the data and input them into the SVM for classification along with the training sample features. The corresponding methods of the three systems (WiGeR, WiFinger, SignFi) shown in this section are the same as above, and there is no difference between supervised and semi-supervised.

#### 5.3.1. Performance of Supervised GANs

**Figure 9:**
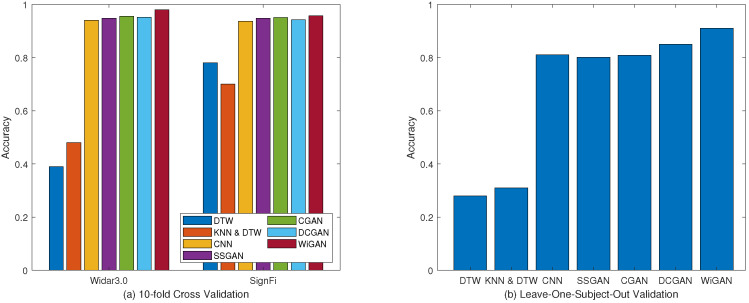
Performance comparison of supervised GANs under the two validation methods. (**a**) 10-fold cross validation. (**b**) Leave-one-subject-out validation.

From Figure 9a, the results show that the accuracy of the four GANs are all over 90%, of which WiGAN has the highest accuracy. In addition, for these GAN-based methods, they are better than previous methods such as DTW and CNN. From this result, the application of GAN to gesture recognition has obtained important gains, which proves that it is feasible to use GAN for data enhancement under small samples. In addition, WiGAN can achieve better or similar results compared with other GAN methods under supervised conditions. This shows that the methods of feature fusion and sample generation have played a role in improving the recognition accuracy. As shown in Figure 9b, for leave-one-subject-out validation, the average recognition accuracy of the four GAN algorithms are 80.1%, 80.9%, 85.1%, and 91%, respectively. Obviously, WiGAN has better robustness for different users.

#### 5.3.2. Performance of Semi-Supervised GANs

Since semi-supervised learning is more suitable for the actual scenario, the recognition accuracy of GANs under semi-supervised conditions is compared. In this experiment, we take some of the labeled data and remove their labels as unlabeled data to simulate a small sample scenario.

**Figure 10:**
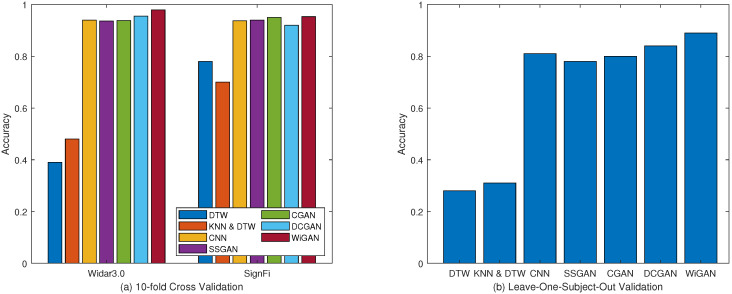
Performance comparison of semi-supervised GANs under the two validation methods. (**a**) 10-fold cross validation. (**b**) Leave-one-subject-out validation.

As shown in Figure 10a, the recognition accuracy of WiGAN is 97.8% in Widar3.0 data, which is between 3% and 4% better than other GAN algorithms. The accuracy of WiGAN is around 95% in SignFi data. As shown in Figure 10b, for leave-one-subject-out validation, WiGAN still has obvious superiority compared with other methods. According to the comparison of supervised learning and semi-supervised learning, DCGAN and WiGAN perform better in Widar3.0 data, but only slightly ahead in SignFi data. From this point of view, even under this condition, WiGAN still has stable recognition accuracy.

On the one hand, we infer that it may be that the method of extracting each layer for data fusion does not perform well when there are many categories. On the other hand, We predict that SVM is difficult to accurately classify extensive samples. When the data distribution is not enough, the accuracy of SVM will increase as the data increases. In the case of large-scale data, increasing the data has little effect on accuracy. However, more and more noise will affect the hyperplane, which is not beneficial for gesture classification. Compared with other methods, using SVM in large-scale data is not only not conducive to improving accuracy, but also greatly increases the complexity.

### 5.4. More Discussions on WiGAN

#### 5.4.1. Performance of Different Classification Methods

**Figure 11:**
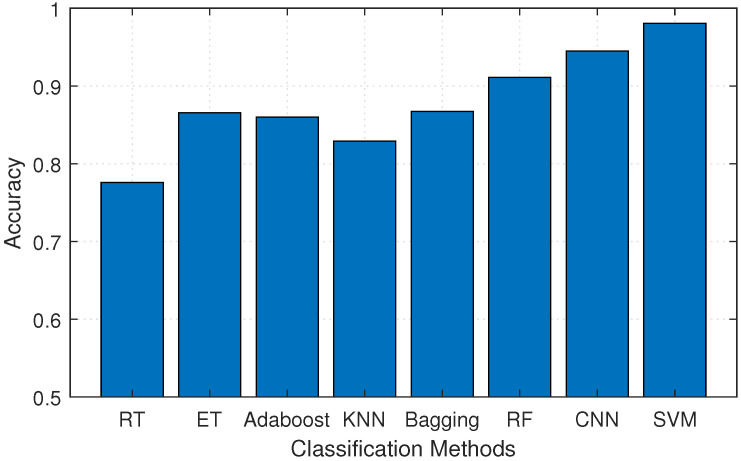
Comparison of recognition accuracy of different classification methods

In order to achieve the highest recognition accuracy, several commonly used classification methods in Widar3.0 data such as Random Tree (RT), Extreme Tree (ET), Adaboost, K-Nearest-Neighbours (KNN), Bagging, Random Forest (RF), CNN, and SVM are compared. The experimental results are shown in Figure 11. It can be found that the average recognition accuracy of SVM is 7% to 20% higher than other methods when performing gesture classification in the small sample conditions, even exceeding CNN. This is because the optimization objective of SVM is to minimize structural risk rather than empirical risk, which reduces the requirements for data size and distribution. With strong generalization ability and fast learning speed, SVM is most suitable for use in our system.

#### 5.4.2. Impact of Number of Links

**Figure 12:**
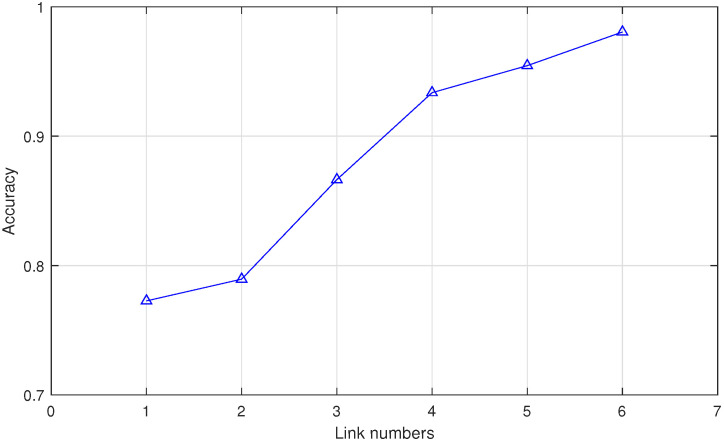
Comparison of the impact of different numbers of links.

The impact of different link numbers on performance is an important discussion in WiFi gesture recognition. To identify more accurately, a total of six links are deployed in Widar3.0. This section studies the impact of the number of different links in Widar3.0 data on the system recognition accuracy. In Figure 12, as the number of links gradually increases, the recognition accuracy gradually increases. The main reason is that the captured information will continue to increase with the increase in the number of links set. When the number of antennas decreases, gesture information in certain directions or positions may be blurred, thereby reducing recognition accuracy.

#### 5.4.3. Impact of CSI Processing

**Figure 13:**
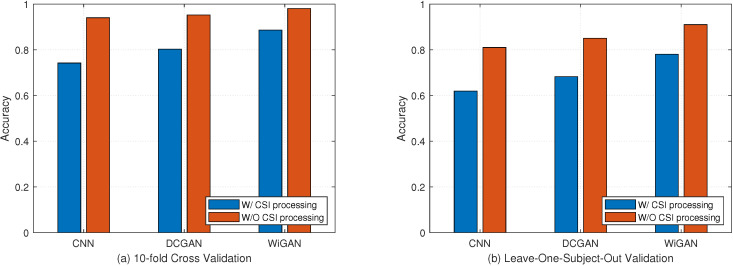
Comparison of the impact of CSI processing modules under the two validation methods. (**a**) 10-fold cross validation. (**b**) Leave-one-subject-out validation.

In WiGAN, the CSI processing module contains four sections: activity detection, interpolation, DWT denoising, and subcarrier selection. The purpose of using the CSI processing module is to convert the raw CSI data to the sanitized CSI amplitude. To better know the performance of the system, the effect of the CSI processing module in Widar3.0 data is discussed in this section. Figure 13a shows the impact of the CSI processing module under 10-fold cross validation. For CNN, DCGAN, and WiGAN systems, the recognition accuracy without using the CSI processing module is only 74.2%, 80.3%, and 88.6%, which is reduced by between 10% and 20% compared to these approaches using the CSI processing module. As shown in Figure 13b, we perform leave-one-out validation on the three approaches. The recognition accuracy is 61.9%, 68.2%, and 78%, which also has a significant degradation compared to these approaches using the CSI processing module. From these two experiments, it can be seen that these approaches of preprocessing CSI have played a key role so that the accuracy can be improved by more than 10%. When CSI preprocessing is not performed, various noises and non-action data will interfere with gesture recognition, and inappropriate subcarrier selection will also lose important gesture feature information, both of which will affect recognition accuracy.

Table 1 shows the computational time of a single gesture. From here, the total time for CSI processing is 0.197 s, which only accounts for 28.26% of the total time. Therefore, from the perspective of cost and benefit, it is cost-effective to add a CSI processing module to the system. More importantly, since the execution time of each gesture exceeds 1 s, WiGAN can provide a real-time action recognition in the process of data measurements.

## 6. Conclusions

In this paper, a GAN-based WiFi gesture recognition system, WiGAN, is proposed to address the problem of the performance degradation caused by small samples and environmental dependence for CSI-based gesture recognition. WiGAN not only enhances the number and diversity of samples, but also incorporates more diverse features, which makes it more successful to conduct the gestures recognition. Experimental results show that the average recognition accuracy of WiGAN can be up to 98% and 95.6% for the Widar3.0 data and SignFi data, respectively. During the experiment, we found that the accuracy of gesture recognition is related to several reasons. First of all, suitable data processing approaches can greatly reduce data noise and establish an excellent foundation for feature extraction. Second, the method of extracting features is the key factor to determine the success of the gesture recognition model. Its design depends on the experimental environment and the collected data. Finally, a robust classifier is the direct cause that affects the accuracy of gesture recognition. In our experiments, we found that similar gestures will affect recognition when the gesture has extensive categories. Thus, for the future work, we will measure data in our environment and expand various activity categories to make our system more versatile.

## Figures and Tables

**Table 1:** Processing time for one gesture.

Activity Detection	Interpolation	DWT Denoising	Subcarrier Selection	Feature Extraction	Classification
0.105 s	0.013 s	0.029 s	0.032 s	0.098 s	0.420 s
